# ECT2 associated to PRICKLE1 are poor-prognosis markers in triple-negative breast cancer

**DOI:** 10.1038/s41416-019-0448-z

**Published:** 2019-04-11

**Authors:** Avais M. Daulat, Pascal Finetti, Diego Revinski, Mônica Silveira Wagner, Luc Camoin, Stéphane Audebert, Daniel Birnbaum, Laurent Kodjabachian, Jean-Paul Borg, François Bertucci

**Affiliations:** 1 Centre de Recherche en Cancérologie de Marseille, Equipe labellisée ‘Cell polarity, Cell Signaling and Cancer’ Ligue 2018, Aix Marseille Université, Inserm, CNRS, Institut Paoli Calmettes, 13009 Marseille, France; 2 Centre de Recherche en Cancérologie de Marseille, Equipe labellisée ‘Predictive oncology’ Ligue 2018, Aix Marseille Université, Inserm, CNRS, Institut Paoli Calmettes, 13009 Marseille, France; 3 Aix Marseille Univ, CNRS, IBDM, Marseille, France; 4 Centre de Recherche en Cancérologie de Marseille, Marseille Proteomics, Aix Marseille Université, Inserm, CNRS, Institut Paoli Calmettes, 13009 Marseille, France

**Keywords:** Gastrulation, Protein-protein interaction networks, Breast cancer

## Abstract

**Background:**

Triple-negative breast cancers (TNBC) are poor-prognosis tumours candidate to chemotherapy as only systemic treatment. We previously found that PRICKLE1, a prometastatic protein involved in planar cell polarity, is upregulated in TNBC. We investigated the protein complex associated with PRICKLE1 in TNBC to identify proteins possibly involved in metastatic dissemination, which might provide new prognostic and/or therapeutic targets.

**Methods:**

We used a proteomic approach to identify protein complexes associated with PRICKLE1. The mRNA expression levels of the corresponding genes were assessed in 8982 patients with invasive primary breast cancer. We then characterised the molecular interaction between PRICKLE1 and the guanine nucleotide exchange factor ECT2. Finally, experiments in *Xenopus* were carried out to determine their evolutionarily conserved interaction.

**Results:**

Among the PRICKLE1 proteins network, we identified several small G-protein regulators. Combined analysis of the expression of PRICKLE1 and small G-protein regulators had a strong prognostic value in TNBC. Notably, the combined expression of *ECT2* and *PRICKLE1* provided a worst prognosis than *PRICKLE1* expression alone in TNBC. PRICKLE1 regulated ECT2 activity and this interaction was evolutionary conserved.

**Conclusions:**

This work supports the idea that an evolutionarily conserved signalling pathway required for embryogenesis and activated in cancer may represent a suitable therapeutic target.

## Background

Triple-negative breast cancer is the most aggressive molecular subtype of breast cancer.^1^ In contrast with mammary cancers of other subtypes (hormone receptor-positive HR+/HER2−, and HER2+), TNBCs do not express hormone receptors nor the HER2 oncogene, and thus are not candidates for hormone and anti-HER2 therapy.^1^ Chemotherapy is the only systemic therapy currently approved for this subtype. However, TNBC is highly invasive with strong metastatic propensity.^1^ We recently identified *PRICKLE1* as a poor-prognosis marker in breast cancer.^2^ PRICKLE1 is a member of a conserved group of proteins involved in planar cell polarity (PCP) pathway.^3^ This pathway is well characterised in epithelial tissue morphogenesis during embryonic development of invertebrates and vertebrates. The organisation of PCP relies on the spatial distribution of proteins at the plasma membrane such as Wnts, Frizzled, Van Gogh, Flamingo, Dishevelled, Diego, and Prickle. In vertebrates, homologous genes are involved in the regulation of convergent-extension (CE) during the early stages of gastrulation which leads to the organisation of cells to generate the head-to-tail axis.^3,4^ Prickle1 plays a pivotal role to regulate PCP in *Drosophila*^5^, as well as CE in Zebrafish^6^ and *Xenopus*.^7^ PRICKLE1 is an evolutionary conserved cytoplasmic protein. It contains a PET domain at the N-terminus followed by three LIM domains and a C-terminal farnesylation site.^8^ Recently, we and others have demonstrated the prominent role of PRICKLE1 during cancer progression.^2,9–11^ PRICKLE1 is a prometastatic protein and regulates oriented cell migration in various cell lines including the MDA-MB-231 prototype TNBC cell line.^2,10^ At the molecular level, PRICKLE1 regulates the subcellular localisation of associated proteins such as VANGL2,^8,12^ RICTOR,^2^ ARHGAP22/24,^10^ and LL5β^11^ to coordinate oriented cellular migration.

Here, we identify the proteome associated to PRICKLE1 in MDA-MB-231 cells. Among the proteins associated to PRICKLE1, our attention was drawn to a large subset of small G-protein regulators. We first show that *PRICKLE1* and its associated proteins are overexpressed in TNBC, and that their upregulation is associated with poor prognosis in this molecular subtype. To further explore the protein complex associated to PRICKLE1, we focused our attention on the Rho-guanylyl exchange factor (GEF) called epithelial cell transforming sequence 2 (ECT2). In non-transformed cells, ECT2 regulates cytokinesis by regulating Rac1 activity.^13–16^ *ECT2* is frequently upregulated in various cancers such as ovarian,^14^ lung,^17^ and breast cancers.^18^ ECT2 promotes Rac1 activity and increases cell growth, invasion, and tumorigenicity.^13,16^ Here, we show that PRICKLE1 is associated with ECT2 to regulate Rac1 activity and that Prickle1 and Ect2 act synergistically during embryonic development. Altogether, these data demonstrate the importance of PRICKLE1 and its associated protein complex as poor-prognosis markers in TNBC and provide evidence that PRICKLE1 may be a suitable therapeutic target for treatment of this aggressive subtype of breast cancer.

## Methods

### Rac1 activity assay

Cells were lysed with ice cold lysis buffer (50 mM Tris, pH 7.6, 150 mM NaCl, 0.1% Triton X-100, 20 mM MgCl_2_ supplemented with protease inhibitor (Sigma)). The supernatant was collected after 10 min of centrifugation at 10,000 ×*g* at 4 °C. Protein concentration was measured from the solubilised fraction and adjusted to 2 mg/mL. Ten per cent of the lysates are conserved as loading controls. One hundred micrograms of GST-CRIB were added to 2 mg of lysate and incubated with rotation during 30 min at 4 °C. Beads were washed with 10 volumes of lysis buffer. Rac-GTP forms were eluted from the beads using 2× Laemmli buffer. Thirty per cent of the sample were ran on 15% SDS-PAGE gel and transferred to PVDF, then blotted with the indicated antibody.

### Breast cancer samples and gene expression profiling

Our institutional series included 353 tumour samples from pre-treatment invasive primary mammary carcinomas either surgically removed or biopsied.^19^ Samples were profiled using Affymetrix U133 Plus 2.0 human microarrays (Santa Clara, CA, USA). The resulting data were pooled with 35 public breast cancer data sets comprising both gene expression profiles generated using DNA microarrays and RNA-Seq and clinicopathological annotations. These sets were collected from the National Center for Biotechnology Information (NCBI)/Genbank GEO, ArrayExpress, European Genome-Phenome Archive, The Cancer Genome Atlas portal (TCGA) databases, and authors’ website (Supplementary Table 1). The final pooled data set included 8982 non-redundant non-metastatic, non-inflammatory, primary, invasive breast cancers.

### Gene expression data analysis

Before analysis, several steps of data processing were applied. The first step was the normalisation of each set separately. It was done in R using Bioconductor and associated packages; we used quantile normalisation for the available processed data from non-Affymetrix-based sets (Agilent, SweGene, and Illumina), and Robust Multichip Average (RMA) with the non-parametric quantile algorithm for the raw data from the Affymetrix-based sets. In the second step, we mapped the hybridisation probes across the different technological platforms represented as previously reported.^20^ When multiple probes mapped to the same GeneID, we retained the most variant probe in a particular data set. We log2-transformed the available TCGA RNA-Seq data that were already normalised. In order to avoid biases related to trans-institutional IHC analyses and thanks to the bimodal distribution of respective mRNA expression levels, the ER, progesterone receptor (PR), and HER2 statutes (negative/positive) were defined on transcriptional data of *ESR1, PGR*, and *HER2*, respectively, as previously described.^21^ The molecular subtypes of tumours were defined as HR+/HER2− for ER-positive and/or PR-positive and HER2-negative tumours, HER2+ for HER2-positive tumours, and triple negative (TN) for ER-negative, PR-negative, and HER2-negative tumours. Next, expression levels of *PRICKLE1* and 10 genes of interest from the protein complex associated with Prickle1 (namely, *ARHGAP21*, *ARGHAP22*, *ARHGAP23*, *ARHGEF2*, *ARHGEF40*, *BCR*, *ECT2*, *IQGAP3*, *MYO9B*, and *STARD13*) were extracted from each of the 36 normalised data sets. Before analysis, gene expression levels were standardised within each data set using the PAM50 luminal A population as reference. This allowed to exclude biases due to laboratory-specific variations and to population heterogeneity and to make data comparable across all sets. *PRICKLE1* and *ECT2* upregulation in a tumour was defined by an expression level above median expression; the other cases being defined as downregulation. GEF/GAP activity was based on metagene approach and computed on the mean of the 10 related genes standardised. GEF/GAP activity “up” was defined by a metagene score value above the global median of the metagene, while a value below the global median was defined as “down”.

### Statistical analysis

Correlations between tumour classes and clinicopathological variables were analysed using the one-way analysis of variance (ANOVA) or the Fisher’s exact test when appropriate. Metastasis-free survival (MFS) was calculated from the date of diagnosis until the date of distant relapse. Follow-up was measured from the date of diagnosis to the date of last news for event-free patients. Survivals were calculated using the Kaplan–Meier method and curves were compared with the log-rank test. The likelihood ratio (LR) tests were used to assess the prognostic information provided beyond that of PRICKLE1 model, GEF/GAP metagene or ECT2 model, assuming a *X*^2^ distribution. Changes in the LR values (LR-Δ*X*^2^) measured quantitatively the relative amount of information of one model compared with another. All statistical tests were two-sided at the 5% level of significance. Statistical analysis was done using the survival package (version 2.30) in the R software (version 2.15.2; http://www.cran.r-project.org/). We followed the reporting REcommendations for tumour MARKer prognostic studies (REMARK criteria).^22^

### *Xenopus* embryo injections, plasmids, RNAs, and Mos

Eggs obtained from NASCO females were fertilised in vitro, dejellied, and cultured as described previously.^23^ Wild-type embryos were obtained using standard methods^24^ from adult animals and staged according to Nieuwkoop and Faber (1994).^25^ Ect2 riboprobe was generated from *Xenopus laevis* full-length Ect2 cDNA, obtained from Dharmacom^TM^ (Plasmid XGC ect2 cDNA, Clone ID: 5083828; pCMV-SPORT6.ccdb). The cDNA was subcloned in pBS-SK vector. For the *Ect2* sense probe, the plasmid was linearised by *Not*I and transcribed with T7 RNA polymerase. For the *Ect2* antisense probe, the plasmid was linearised by *Eco*RV and transcribed with T3 RNA polymerase. Synthetic capped mRFP mRNA was produced using Ambion mMESSAGE mMACHINE Kit. pCS2-mRFP was linearised with *Not*I and mRNA was synthesised with Sp6 polymerase. About 0.5 ng of mRFP capped mRNA was used as injection control and tracer.

Morpholino antisense oligonucleotides (MO) were obtained from Genetools with the following: Prickle1 (Pk1) 5′-CCTTCTGATCCATTTCCAAAGGCAT-3′;^26^ ECT2 5′-TACTGGGAGAGCCATGTTTGATTT-3′. Embryos at the two-cell stage were injected in each blastomere with various doses of MOs. Embryos were cultured in modified Barth’s solution until stage 28, when they were photographed.

Extended material methods including chapter about: -Plasmid constructs and reagents, tissue culture and transfection, -Immunopurification, -Affinity purification, -immunoprecipitation and western blot, -Mass spectrometry analysis. -Protein identification, -In situ hybridisation (ISH) are available in Supplementary material and methods.

## Results

### Mass spectrometry analysis of the PRICKLE1 complex shows that PRICKLE1 is associated with small G-protein regulators

**Fig. 1:**
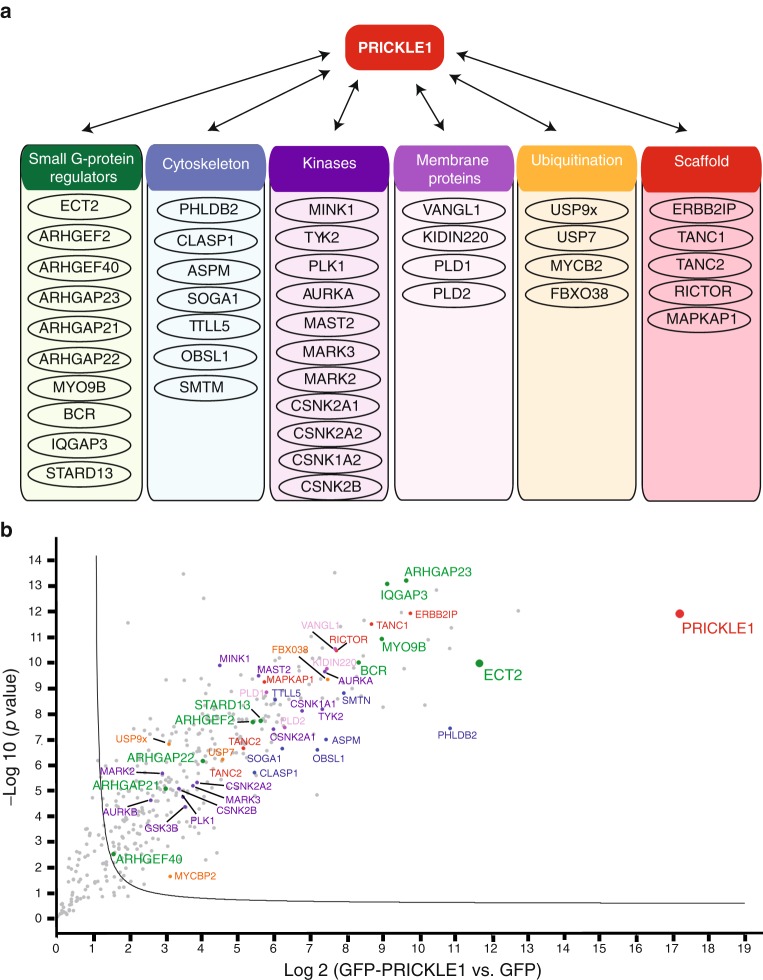
Mass spectrometry analysis of the PRICKLE1 protein complex from a TNBC cell line. **a** Schematic representation of the proteins associated to PRICKLE1 identified by mass spectrometry analysis from MDA-MB-231 cell extracts. Proteins have been classified following their function in several groups: Small G-proteins regulators, cytoskeleton-associated, kinases, membrane proteins, proteins involved in ubiquitination, scaffold proteins, and others. **b** Volcano plot showing the significance two-sample *t*-test (−Log *p* value) vs. fold-change (Log2 (GFP-PRICKLE1 vs. GFP as control)) on the *y* and *x* axes, respectively. The full line is indicative of protein hits obtained at a permutation false discovery rate of 1% (pFDR). Data results from two different experiments processed three times. PRICKLE1 (the bait) is represented in red and ECT2, one of the most abundant PRICKLE1-associated partners, is represented in green

We and others have shown that PRICKLE1 contribute to cancer cell dissemination in various cancers.^2,9–11^ To investigate the molecular mechanisms underlying the role of PRICKLE1 in tumorigenicity, and notably cell motility and dissemination, we generated a stable cell line expressing GFP-PRICKLE1 in the highly invasive MDA-MB-231 TNBC cell line. To identify protein complexes associated to PRICKLE1 in these cells, we performed anti-GFP immunoprecipitation followed by mass spectrometry analysis. We identified previously known PRICKLE1 interactors such as VANGL1, MINK1, RICTOR, LL5β, PLK1, and USP9x, validating our approach (Fig. 1a). Cell migration is a complex and dynamic process that involves continuous remodelling of the cellular architecture and relies on spatiotemporal modulation of signalling networks including Rho-family GTPases. Our attention was drawn to the large number of regulators of Rho-family GTPases such as Rac1, Rho, and Cdc42 (Fig. 1b), known to be involved in the regulation of cell motility, and considered as interesting drug targets to prevent cancer dissemination.

### Prognostic value of PRICKLE1-interacting small G-protein regulators in TNBC

**Fig. 2:**
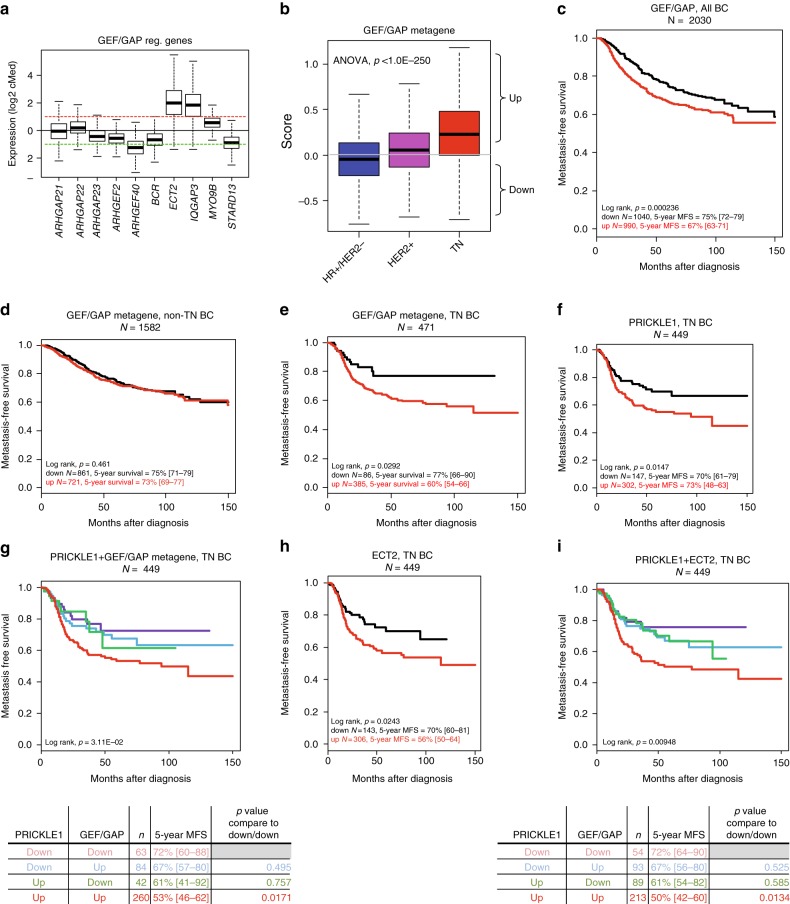
Prognostic value of PRICKLE1-interacting small G-protein regulators in TNBC and cooperation between *PRICKLE1* and *ECT2* as poor-prognosis markers. **a** Boxplot of GEF/GAP regulators expression across breast cancers. **b** Boxplot of GEF/GAP regulators expression across triple negative (TN) vs. HR+/HER2− or HER2+ breast cancers. **c** Kaplan–Meier curves of metastasis-free survival among breast cancers patients according to overexpression (up) vs. underexpression (down) of GEF/GAP metagene mRNA. **d** Kaplan–Meier curves of metastasis-free survival among non-TNBC patients for GEF/GAP metagene mRNA expression. Kaplan–Meier curves of metastasis-free survival among TNBC patients for **e** GEF/GAP metagene mRNA expression, **f** *PRICKLE1* mRNA expression, **g** *PRICKLE1* mRNA and GEF/GAP metagene expression, **h** *ECT2* mRNA expression, and **i** *PRICKLE1* and *ECT2* mRNA expression

Based on these proteomic data describing the protein complex associated to PRICKLE1, we focused our attention on the 10 regulators of small G-proteins (i.e. Rho-GEF and Rho-GAP) that were identified, including ARHGAP21, ARGHAP22, ARHGAP23, ARHGEF2, ARHGEF40, BCR, ECT2, IQGAP3, MYO9B, and STARD13. We assessed the mRNA expression level of the corresponding genes in a retrospective series of 8982 clinically annotated patients with invasive primary breast cancer collected from several public databases (Table S1). Within these 10 genes, *ECT2*, *IQGAP3*, and *MYO9B* were the most overexpressed in tumours compared to normal breast tissues (Fig. 2a), whereas *ARHGEF40* and *STARD13* showed the lowest expression levels. We built a metagene including these 10 genes (GEF/GAP metagene) and compared its expression level in three molecular subtypes of breast cancer (HR+/HER2−, HER2+, and TN). The metagene was significantly upregulated in the TN subtype compared to the two other subtypes (*p* < 1.0 × 10^−250^, ANOVA) (Fig. 2b).

We then searched for correlations between the GEF/GAP metagene expression (as a binary variable) and the clinicopathological features of samples, including MFS. Within the 8982 breast cancer samples analysed, 4491 tumours (50%) showed metagene upregulation when compared with normal breast (ratio T/NB ≥2; “metagene-up” group), and 4.491 (50%) showed metagene downregulation (ratio <2; “metagene-down” group) (Table 1). We found significant correlations between the metagene status and patients’ age (*p* < 0.001), grade (*p* < 0.001), ER (*p* < 0.001), PR (*p* < 0.001), and HER2 (*p* = 0.012) statutes and with molecular subtypes of breast cancer. MFS data were available for 2030 patients: the 5-year MFS was 75% (95% Cl, 72–79) in the “metagene-down” group vs. 67% (95% Cl, 63–71) in the “metagene-up” group (*p* = 0.00023, log-rank test; Fig. 2c). In fact, this prognostic correlation was only observed in TNBC patients, and not in the non-TNBC ones (*p* = 0.461, log-rank test; Fig. 2d). In TNBC patients, the 5-year MFS was 77% (95% Cl, 66–90) in the “metagene-down” group vs. 60% (95% Cl, 54–66) in the “metagene-up” group (*p* = 0.029, log-rank test; Fig. 2e).

**Table 1:** Clinicopathological characteristics of samples in the whole cohort and in subgroup defined according to the GEF/GAP metagene-based classifier

Characteristics	*N* (%)	GEF/GAP metagene		*p* Value
		Down	Up	
Age at diagnosis (years)				3.46E-14
≤50	2540 (36%)	1112 (32%)	1428 (40%)	
>50	4488 (64%)	2388 (68%)	2100 (60%)	
Pathological type				1.21E-02
Ductal	3979 (79%)	1998 (77%)	1981 (80%)	
Lobular	498 (10%)	263 (10%)	235 (10%)	
Other	574 (11%)	325 (13%)	249 (10%)	
Pathological tumour size (pT)				0.133
pT1	2113 (38%)	1100 (39%)	1013 (36%)	
pT2	2923 (52%)	1439 (51%)	1484 (53%)	
pT3	595 (11%)	304 (11%)	291 (10%)	
Pathological axillary node status (pN)				0.239
0	3446 (56%)	1741 (56%)	1705 (55%)	
1	2743 (44%)	1344 (44%)	1399 (45%)	
Pathological grade				1.00E-06
1	721 (11%)	442 (14%)	279 (9%)	
2	2573 (41%)	1478 (48%)	1095 (34%)	
3	2986 (48%)	1181 (38%)	1805 (57%)	
ER mRNA status				2.29E-153
Negative	2764 (31%)	811 (18%)	1953 (43%)	
Positive	6218 (69%)	3680 (82%)	2538 (57%)	
PR mRNA status				8.62E-53
Negative	4670 (52%)	1976 (44%)	2694 (60%)	
Positive	4255 (48%)	2489 (56%)	1766 (40%)	
ERBB2 mRNA status				0.00021
Negative	7884 (88%)	4000 (89%)	3884 (86%)	
Positive	1098 (12%)	491 (11%)	607 (14%)	
Molecular subtype				1.00E-06
HR+/HER2−	5929 (66%)	3532 (79%)	2397 (54%)	
HER2+	1098 (12%)	491 (11%)	607 (14%)	
TN	1936 (22%)	463 (10%)	1473 (33%)	
Metastatic relapse				6.81E-06
No	3127 (77%)	1606 (80%)	1521 (74%)	
Yes	923 (23%)	396 (20%)	527 (26%)	
Follow-up, median (range)	42 (1–232	38 (1–221)	37 (1–232)	0.48
5-year MFS, % [95% CI]	71% [69–74]	75% [72–79]	67% [64–71]	5.09E-04

*GEF* Rho-guanylyl exchange factor, *GAP* GTPase-activating proteins, *ER *Oestrogen receptors, *PR* progesterone receptor, *MFS* metastasis-free survival

### Cooperation between PRICKLE1 and ECT2 as poor-prognosis markers in TNBC

We have previously shown that *PRICKLE1* upregulation is associated with poor MFS in basal breast cancer,^2^ a molecular subtype mainly composed of TNBC. In the present series of TNBC, we confirmed that *PRICKLE1* upregulation was associated with shorter MFS, with 70% 5-year MFS (95% Cl, 61–79) vs. 55% (95% Cl, 48–63) in the *PRICKLE1*-down group and the *PRICKLE1*-up group, respectively (*p* = 0.0147, log-rank test) (Fig. 2f). Since *PRICKLE1* and the 10 genes of the metagene interact together, we tested whether their interaction had any prognostic value. First, we analysed the combination of the metagene expression and *PRICKLE1* expression. Interestingly, patients with upregulation of both markers displayed shorter 5-year MFS (53%, 95% Cl, 46–62) than patients without upregulation of both markers (72%, 95% Cl 60–88; *p* = 0.017, log-rank test), whereas patients with intermediate status (upregulation and downregulation, and vice versa) showed intermediate 5-year MFS not significantly different from the same patients (*p* = 0.757 and *p* = 0.495, respectively, log-rank test; Fig. 2g). These data suggest that metagene expression and *PRICKLE1* expression might provide a complementary prognostic value. This complementarity between the two prognostic variables was tested in TNBC patients using the likelihood ratio (LR) test. As shown in Table 2a, the metagene added prognostic information to that provided by *PRICKLE1* expression (LR-Δ*X*^2^ = 2.75, *p* = 0.097).

**Table 2:** Comparison of the prognostic value of different models based on gene expression in TNBC

A *PRICKLE1 & GEF/GAP*			
MFS,TN BC	Statistic		*p* value
PRICKLE1	LRX²	6.23	1.25E-02
PRICKLE1 + GEF/GAP	LRX²	8.98	1.12E-02
GEF/GAP + PRICKLE1 vs. PRICKLE1	ΔLRX²	2.75	0.097
B *PRICKLE1 & ECT2*			
MFS,TN BC	Statistic		*p* value
PRICKLE1	LRX²	6.23	1.25E-02
PRICKLE1 + ECT2	LRX²	11	4.14E-03
ECT2 + PRICKLE1 vs. PRICKLE1	ΔLRX²	4.74	2.90E-02

Second, because ECT2 was one of the most prominent hit identified by mass spectrometry analysis (Fig. 1b) and the gene most overexpressed in TNBCs among members of the metagene (Fig. 2a), we investigated whether *ECT2* expression alone (without the nine other genes of the metagene) would be sufficient to improve the prognostic value of *PRICKLE1* expression in TNBC patients. As shown in Fig. 2h, patients with *ECT2* upregulation displayed shorter 5-year MFS (56%, 95% Cl 50–64) than patients without upregulation (70%, 95% Cl 60–81; *p* = 0.0243, log-rank test). More interestingly, ECT2 expression status increased the prognostic value of *PRICKLE1* expression when combined. Patients with upregulation of both genes displayed 50% 5-year MFS (95% Cl, 46–62) vs. 67% for patients with intermediate status (up and down, and vice-versa) vs. 76% (95% Cl, 64–90) for patients without upregulation of both markers (*p* = 0.0134, log-rank test; Fig. 2i). The model comparison (Table 2b) showed that such *ECT2* prognostic information added to that of *PRICKLE1* expression was statistically significant (LR-Δ*X*^2^ = 4.74, *p* = 0.029), indicating that *ECT2* expression improved the prognostic value of *PRICKLE1* expression in TNBC.

### PRICKLE1 binds to ECT2 through its PET domain and modulates Rac1 activity

**Fig. 3:**
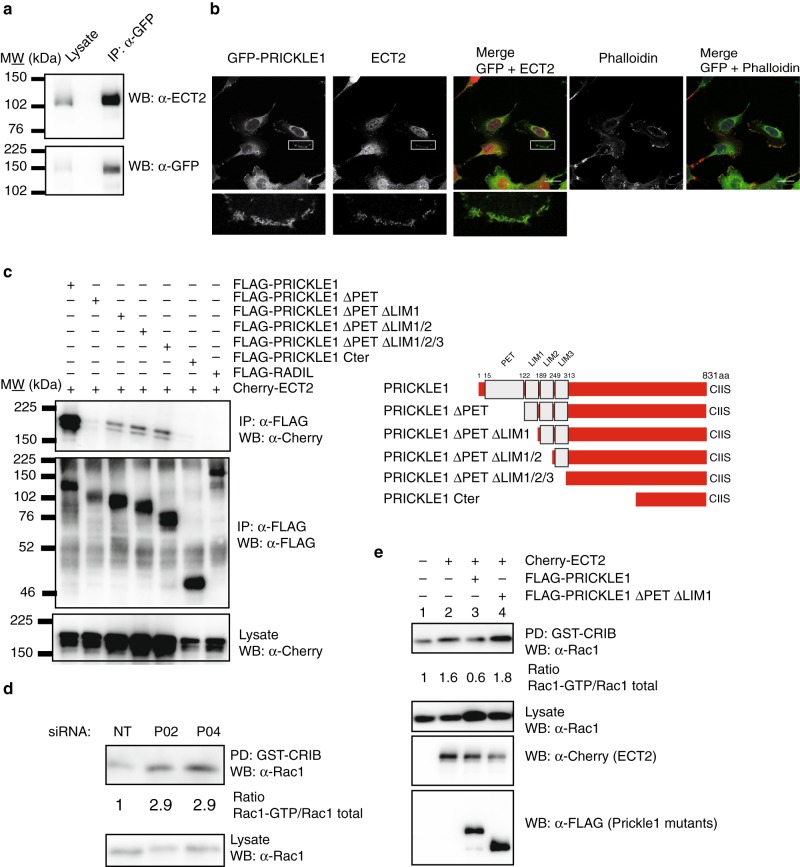
PRICKLE1 is associated to the Rho-GEF ECT2 and controls its activity. **a** Immunopurification of GFP-PRICKLE1 from MDA-MB-231 cell lysate using GFP nanobodies coupled to sepharose beads allows the identification of ECT2 associated to PRICKLE1. **b** Immunofluorescence of MDA-MB-231 cells stably expressing GFP-PRICKLE1 shows that ECT2 (endogenous) is colocalised with PRICKLE1 and enriched in actin structures within the lamellipodia. **c** Mapping of the PRICKLE1 domain needed for interaction with ECT2. HEK293T cells were co-transfected with the indicated forms of PRICKLE1 (see on the left for topology details) and mCherry-ECT2. After FLAG immunopurification, presence of ECT2 is detected using anti-mCherry antibody. **d** Downregulation of PRICKLE1 expression using siRNA targeting *PRICKLE1* shows an increase of Rac activity in MDA-MB-231 cells. **e** PRICKLE1 modulates ECT2 activity. Using HEK293T cells, we expressed or co-expressed ECT2 with full length or a deleted version of PRICKLE1 lacking its domain of interaction with ECT2. Overexpression of ECT2 leads to an increase in Rac activity which was inhibited when PRICKLE1 is co-expressed. Co-expression of a mutant form of PRICKLE1 did not modify the gain of function observed by ECT2 overexpression

We then investigated the molecular mechanisms potentially associated to this cooperation of PRICKLE1 and ECT2 expressions to confer poor prognosis. ECT2 is a Rho-GEF and acts by exchanging GDP to GTP on the small GTPases, RhoA, Rac1, and Cdc42.^27^ To confirm our mass spectrometry analysis, we immunoprecipitated GFP-PRICKLE1 stably expressed in MDA-MB-231 cells using GFP-targeted antibody and assessed the presence of ECT2 associated to PRICKLE1 by western blot analysis complex (Fig. 3a). We confirmed that ECT2 is associated with PRICKLE1 in MDA-MB-231 cells. We further showed that ECT2 colocalises in actin-enriched structures of lamellipodia along with PRICKLE1 using MDA-MB-231 stably expressing GFP-PRICKLE1 (Fig. 3b). We next mapped the domain of interaction between PRICKLE1 and ECT2. We thus generated deleted versions of PRICKLE1 that lack the PET and/or the LIM domains and a construct encompassing the PRICKLE1 C-terminal region. We co-transfected HEK293T cells with the indicated FLAG tagged PRICKLE1 mutants with mCherry-ECT2. After FLAG immunoprecipitation, we assessed the presence of mCherry-ECT2 by western blot analysis. We observed that the PET domain of PRICKLE1 was required for the formation of the PRICKLE1-ECT2 protein complex (Fig. 3c).

We further assessed PRICKLE1 contribution to Rac activity. We used previously characterised siRNAs^2^ to specifically downregulate PRICKLE1 expression in MDA-MB-231 cells. We observed that PRICKLE1 modulated Rac1 activity, suggesting a prominent role of PRICKLE1 in the regulation of Rho-GEF and Rho-GAP (Fig. 3d). We next set up an assay to monitor the role of PRICKLE1 on ECT2 Rho-GEF activity. We expressed mCherry-ECT2 in HEK293T cells and observed an increase of active Rac1 (lane 2). However, when FLAG-PRICKLE1 was co-expressed with mCherry-ECT2, we observed an inhibitory effect of PRICKLE1 (lane 3). This observation was confirmed by the co-expression of a PRICKLE1 mutant lacking the PET and LIM1 domains which was unable to bind ECT2 and did not affect the gain of activity of ECT2 in our system (Fig. 3e, **lane 4**). Altogether, our data suggest that PRICKLE1 is associated with ECT2 in actin-rich structures within the lamellipodia of the cells in order to modulate the activity of ECT2 on Rac1.

### Prickle1 and Ect2 functionally interact in *Xenopus* during embryonic development

**Fig. 4:**
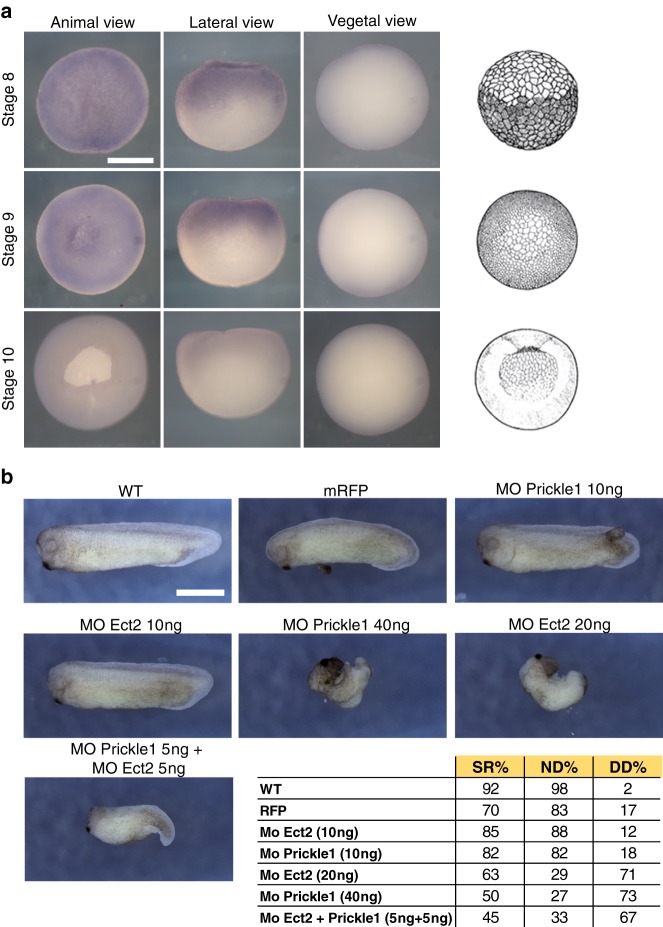
Prickle1 and Ect2 functionally interact in *Xenopus* during embryonic development. In situ hybridisation against *ect2* transcripts at stage 8, 9, and 10. *ect2* RNA is detectable in the animal pole (animal view and lateral view) but not in the vegetal pole (vegetal view) at stages 8 and 9, but no longer at stage 10. Schematic representations of embryos at the stages analysed are shown on the right. **b** Embryos at two-cell stage were injected into two blastomeres with Prickle1 and Ect2 MOs as indicated. In all cases 0.5 ng of *mRFP* mRNA was injected as control and tracer. Suboptimal doses (10 ng) of either MO did not cause CE problems. However, when both Prickle1 and Ect2 MOs were co-injected at suboptimal doses (5 ng each), embryos displayed CE problems at a rate comparable to high doses of each MO injected separately (40 ng Prickle1-MO or 20 ng Ect2-MO). A total of 60 embryos per condition was analysed in two independent experiments. Pictures illustrate representative phenotypes. SR survival rate, ND percentage of surviving embryos developing normally, CED, percentage of surviving embryos showing convergent-extension defects. Scale bars: **a** = 0.25 mm; **b** = 0.5 mm

PRICKLE1 is an evolutionarily conserved protein and plays a pivotal role during gastrulation to modulate CE movements, which are crucial to shape the body plan.^7,28^ To test whether Ect2 is required for the previously characterised function of Prickle1 during CE, we first compared and analysed the RNA-seq profile of *prickle1* and *ect2* reported on the public XenBase repository^29^ (data not shown). We noticed a sharp peak of zygotic *ect2* expression at stage 9, which decreases abruptly at stage 10, just before gastrulation and CE movements take place. Zygotic *prickle1* expression also begins to increase at stage 9, reaching a maximum at stage 12 (mid gastrula), and gradually decreasing until the end of neurulation. We next performed in situ hybridisation and detected expression of *ect2* RNA in the animal hemisphere up until stage 9 (Fig. 4a). Thus, *ect2* transcription appears to terminate when *prickle1* transcription starts. However, inspection of genome-wide proteomic data^30^ indicated that Ect2 protein levels were maintained during gastrulation, suggesting that Ect2 could cooperate with Prickle1 to regulate morphogenetic movements. To test this hypothesis, we performed Prickle1 and Ect2 knockdown through antisense morpholinos (MO) injections and assessed CE problems (Fig. 4b). Injection of 40 ng Prickle1 MOs led to CE defects in 73% of embryos, in comparison to non-injected embryos (98%) or embryos injected with RFP as control (83%). These data are consistent with previously published results.^7,12^ We then injected 20 ng of MO targeting Ect2 and we observed CE problems at a rate of 71%, phenocopying the effect observed with Prickle1 MOs with narrower and shorter embryos at tailbud stage 28. We then defined subthreshold doses of individual Mo-Prickle1 ( ≤10 ng) and Mo-Ect2 (≤10 ng) that yielded moderate CE defects in this assay when injected separately into two blastomeres at two-cell stage (18% and 12% CE defects, respectively). In contrast, co-injecting both MOs at subthreshold doses caused strong disruption of CE movements (67%), suggesting that Prickle1 and Ect2 functionally interact during *Xenopus* embryonic development.

## Discussion

We and others have previously demonstrated the prominent role of PRICKLE1 during cancer progression.^2,9–11^ In this study, we identified the protein complex associated to PRICKLE1 and we evaluated the impact of PRICKLE1 and its associated protein complex in TNBC. Our results show that PRICKLE1 acts as a scaffold protein due to the large number of associated proteins with enzymatic activity. Among the PRICKLE1-associated proteins, we focused our attention on small G-protein regulators since their impact on cell motility and cancer cell dissemination has been well characterised.^31–33^ Exploiting our transcriptomic breast cancer database, we showed that this subset of genes is upregulated in TNBC. Among this group of genes, we identified *ECT2* as the most prominent contributor to *PRICKLE1* prognostic value. Indeed, TNBC patient with upregulated expression of both *PRICKLE1* and *ECT2* expression had a shorter MFS than other patients. We further characterised the PRICKLE1 and ECT2 interaction and showed that PRICKLE1 controls ECT2 function on Rac1 activation. We finally defined that Prickle1 and Ect2 interaction was evolutionary conserved, since both proteins contribute to *Xenopus* embryonic development and are involved in CE movements.

Among breast cancers, TNBC are considered the most aggressive form and no targeted therapy is currently available due to a lack of specific targets.^1^ Here, we show that *PRICKLE1* is overexpressed in TNBC and is a poor-prognosis marker. PRICKLE1 is a protein highly regulated by post-translational modifications, particularly through ubiquitination/deubiquitination. PRICKLE1 is indeed the target of SMURF1, an ubiquitin ligase, which allows its rapid degradation,^34^ but is also protected from degradation by USP9x which de-ubiquitinates the protein.^35^ Interestingly USP9x is also upregulated in several cancers and is considered as a poor-prognosis marker.^36^ PRICKLE1 is also regulated through phosphorylation by the serine/threonine kinase called MINK1, which promotes its function, its membrane localisation and association with signalling molecules.^12^ Together, this shows that PRICKLE1 is a pivotal protein in cancer cell dissemination and a candidate target for setting up novel therapeutic strategies.

During developmental processes and cancer progression, PRICKLE1 is required for oriented cell migration.^2,9,11,37^ At the molecular level, we and others have shown that PRICKLE1 functions to localise VANGL at the plasma membrane,^8,12^ LL5β at the + ends of the microtubules,^11^ and to restrict localisation of Rho-GAP at the edge of the migrating cancer cells.^10^ PRICKLE1 also regulates spatial localisation of several active proteins such as mTORC2 to allow local activation of Akt at the leading edge of migrating cells,^2^ PHLDB2 to disassemble focal adhesions^11^ and to restrict RhoA activity by regulating subcellular localisation of Rho-GAP.^10^ Together the contribution of PRICKLE1 to localisation of its interacting partners allows the cells to coordinate cellular movements and promote directed cell migration. Here we show that PRICKLE1 also contributes to regulating the activity of ECT2, a GEF for Rac1, which is essential for cell motility.

ECT2 is a Rho-GEF controlling Rac1 activity.^13^ Although ECT2 has been extensively studied for its role in the nucleus and during cytokinesis, reports have shown that ECT2 can also be localised in the cytoplasm of cancerous cells.^16^ We observed that ECT2 is localised in actin-rich structures within the lamellipodia. As described for other PRICKLE1 interactors, PRICKLE1 might contribute to ECT2 spatial localisation in order to modulate its Rac activity. Moreover, our data show that overexpression of ECT2 in HEK293T cells contributes to an increase of Rac activity, and that PRICKLE1 overexpression leads to a decrease of this gain of function, suggesting an inhibitory role of PRICKLE1 on ECT2 activity. Altogether, this depicts PRICKLE1 as a master regulator of localised expression and regulation of signalling events in migratory cancer cells.

Our data also identified a role for the PET domain of PRICKLE1, as ECT2 is to date the only protein identified to be associated with this domain. At the molecular level, it has been shown that PRICKLE1 exists in an open and closed conformation.^38^ It has been suggested that in the closed conformation, the three LIM domains of PRICKLE1 mask the PRICKLE1 PET domain. In an open conformation, the PET domain is unmasked, thus activating PRICKLE1. We can speculate that the interaction between PRICKLE1 and ECT2 can be modulated by switching between these two conformations as a molecular mechanism for PRICKLE1 activation.

Finally, our study identified that ECT2 is required for *Xenopus* embryonic development. Prickle1 has been extensively characterised for its contribution during CE^6,7^ movements and has been shown to be asymmetrically distributed within the cells in order to organise their movement.^39,40^ A previous study indicated that *Prickle1* mRNA accumulates within the blastopore lip from the onset of gastrulation.^41^ Here, we show that *ect2* mRNA and presumably Ect2 protein are expressed prior to and in a broader pattern than Prickle1.^41^ Knockdown experiments strongly suggest that Prickle1 and Ect2 act together to allow convergence-extension movements during gastrulation. Altogether, our data support the view that Ect2 might represent a permissive factor for Prickle1 activity. This study demonstrates the importance of the evolutionarily conserved interaction between Prickle1 and Ect2, which appears to be reactivated during tumorigenesis to promote cancer cell dissemination and metastasis.

## Supplementary information

Supplementary Table 1

Supplementary Material and methods

## Data Availability

The mass spectrometry proteomics data, including search results, will be deposited to the ProteomeXchange Consortium (www.proteomexchange.org)^42^ via the PRIDE partner repository with the data set identifier PXD011253.
